# An alternative model of maternity care for low‐risk birth: Maternal and neonatal outcomes utilizing the midwifery‐based birth center model

**DOI:** 10.1111/1475-6773.14222

**Published:** 2023-09-10

**Authors:** Jacqueline Wallace, Lauren Hoehn‐Velasco, Ellen Tilden, Bryan E. Dowd, Steve Calvin, Diana R. Jolles, Jennifer Wright, Susan Stapleton

**Affiliations:** ^1^ American Association of Birth Centers Research Committee Perkiomenville Pennsylvania USA; ^2^ Department of Economics, Andrew Young School of Policy Studies Georgia State University Atlanta Georgia USA; ^3^ Nurse‐Midwifery Department, School of Nursing Oregon Health and Science University Portland Oregon USA; ^4^ Department of OBGYN, School of Medicine Oregon Health and Science University Portland Oregon USA; ^5^ Division of Health Policy and Management, School of Public Health University of Minnesota Minneapolis Minnesota USA; ^6^ Department of Obstetrics, Gynecology and Women's Health University of Minnesota Medical School Minneapolis Minnesota USA; ^7^ Clinical Faculty Frontier Nursing University Tucson Arizona USA

**Keywords:** birth center, community birth, low‐risk birth, maternity care models, midwifery‐led birth center, propensity score weighting

## Abstract

**Objective:**

To assess key birth outcomes in an alternative maternity care model, midwifery‐based birth center care.

**Data Sources:**

The American Association of Birth Centers Perinatal Data Registry and birth certificate files, using national data collected from 2009 to 2019.

**Study Design:**

This observational cohort study compared key clinical birth outcomes of women at low risk for perinatal complications, comparing those who received care in the midwifery‐based birth center model versus hospital‐based usual care. Linear regression analysis was used to assess key clinical outcomes in the midwifery‐based group as compared with hospital‐based usual care. The hospital‐based group was selected using nearest neighbor matching, and the primary linear regressions were weighted using propensity score weights (PSWs). The key clinical outcomes considered were cesarean delivery, low birth weight, neonatal intensive care unit admission, breastfeeding, and neonatal death. We performed sensitivity analyses using inverse probability weights and entropy balancing weights. We also assessed the remaining role of omitted variable bias using a bounding methodology.

**Data Collection:**

Women aged 16–45 with low‐risk pregnancies, defined as a singleton fetus and no record of hypertension or cesarean section, were included. The sample was selected for records that overlapped in each year and state. Counties were included if there were at least 50 midwifery‐based birth center births and 300 total births. After matching, the sample size of the birth center cohort was 85,842 and the hospital‐based cohort was 261,439.

**Principal Findings:**

Women receiving midwifery‐based birth center care experienced lower rates of cesarean section (−12.2 percentage points, *p* < 0.001), low birth weight (−3.2 percentage points, *p* < 0.001), NICU admission (−5.5 percentage points, *p* < 0.001), neonatal death (−0.1 percentage points, *p* < 0.001), and higher rates of breastfeeding (9.3 percentage points, *p* < 0.001).

**Conclusions:**

This analysis supports midwifery‐based birth center care as a high‐quality model that delivers optimal outcomes for low‐risk maternal/newborn dyads.

**What is known on this topic**

- The United States has the worst maternal outcomes of any high‐income country, and improving the maternity care system is challenging given the divergent needs of those with high‐risk versus low‐risk pregnancies.
- The midwifery‐based birth center model of maternity care centers midwifery principles, with birth occurring in a nonmedicalized environment, and evidence from outside the United States supports improved outcomes for low‐risk pregnancies.
- Research conducted in the United States on this model of maternity care, typically conducted using either birth certificate data or perinatal data registries, has resulted in conflicting results regarding clinical outcomes.

**What this study adds**

- This study used robust statistical methods, a perinatal data registry, and birth certificate data to evaluate a national sample of US births managed in the midwifery‐based birth center model.
- The use of these data sources allowed for evaluation of the full model of midwifery‐based birth center care, beyond simply place of birth or birth attendant.
- This analysis found that women and their neonates at low risk for perinatal complications who received care in the midwifery‐based birth center model experienced improved outcomes.

## 1 INTRODUCTION

The United States has the worst maternal outcomes of any wealthy country, despite spending more per childbirth episode than any other nation in the world.^1^, ^2^ Like many countries, the US faces a dual burden of “too little too late” and “too much too soon.” As defined by Miller and colleagues, too little too late occurs when limited supplies and infrastructure prevent the provision of quality obstetric care. Conversely, too much too soon occurs when the over‐medicalization that often characterizes modern obstetric practice leads to inappropriate or excessive interventions.^3^

While the US level of intervention frequently delivers high‐quality care to those with perinatal complications, it is poorly matched to the needs of low‐risk maternal/newborn dyads.^3^, ^4^, ^5^ The majority of pregnant women in the US remain low risk throughout pregnancy, delivery, and postpartum.^6^ Given the potential harms created by nonindicated interventions,^3^, ^4^, ^5^ it is important to seek ways to improve care for maternal/newborn dyads who are at low risk for perinatal complications. Using a large national sample of women at low risk for perinatal complications, we compare key birth outcomes of a cohort who received midwifery‐based birth center care versus those who received hospital‐based usual care to evaluate models of care for low‐risk pregnancies.

The study of low‐risk childbirth has increased over the last several decades, with particular attention to evaluating the impact of care provider and birth setting on outcomes. Much of the research, however, has considered these factors separately.^7^, ^8^ The current study builds upon existing research by using perinatal registry data, birth certificate data, and robust statistical analyses to allow for a more comprehensive evaluation of the midwifery‐based birth center model as an integrated model of maternity care.

While the term “midwifery‐based birth center care” clearly refers to birth location, the model goes beyond birth setting. The midwifery‐based birth center model includes pregnancy, birth, and postpartum care, and the birth centers in our study include a variety of birth settings. In this care model, birth typically occurs in a free‐standing birth center, which is a nonhospital facility that provides a safe, home‐like environment for low‐risk childbirth.^9^ Midwifery‐based birth centers are integrated into the broader US maternity care system. Transfers may occur from birth center care to hospital‐based care at any point due to patient choice or increasing medical risk. In most cases of transfer, hospital providers take over medical management and the patient transitions from the midwifery‐based birth center model to the hospital‐based usual care model. We use the phrase “midwifery‐based birth center” because the majority of birth centers are staffed by midwives.^10^ The model of care, however, can be provided by physicians or midwives.^11^

The US differs from most other high‐income countries in having several distinct training and credentialing mechanisms for midwives. Certified nurse midwives (CNMs) and certified midwives (CMs) receive equivalent graduate‐level education, have the same scope of practice, and have the same certifying board, although CMs do not have a nursing degree while CNMs do.^12^ Direct entry midwives, such as certified professional midwives (CPMs) have a competency‐based approach to training and certification, either through apprenticeship or through a CPM educational program. CPMs spend a median of 3 years in training prior to attending births as primary midwife.^13^ State regulation of midwifery practice varies widely; CNMs are licensed in all 50 states, CMs in 7 and CPMs in 34. The education and training requirements of CNMs, CMs, and CPMs align with the International Confederation of Midwives Global Standards for Midwifery Education.^12^, ^13^, ^14^ There are approximately 12,000 CNMs, 2300 CPMs, and 100 CMs in the United States.^12^ Ninety‐four percent of CNM‐attended births occur in hospitals,^15^ while the majority of CPM‐attended births are home births.^13^ Both CNM/CMs and CPMs provide care in the midwifery‐based birth center model of care; a recent American Association of Birth Centers (AABC) survey of birth centers in the US found that approximately 44% of birth centers had exclusively CNM/CM providers, 34% exclusively CPM providers, and 22% had a mix of all three types.^10^

Fewer than 1% of all pregnant women in the US receive midwifery‐based birth center care.^16^ In this model, care is based on midwifery principles, which view pregnancy through a wellness lens, with a focus on optimizing normal physiologic birth and “the art of doing nothing well,” or continual vigilance and support with intervention when beneficial.^17^ Core tenets include respecting cultural differences, supporting client autonomy, and limiting interventions unless medically necessary.^9^, ^18^, ^19^ The care processes of the midwifery‐based birth center model of care are distinct from the hospital‐based usual care model of maternity care. Prenatal visits are generally three to four times longer, with an emphasis on education, empowerment, and shared decision‐making.^18^, ^20^ Intrapartum care processes are also distinct; in the birth center model, labor occurs in a nonmedicalized environment that encourages freedom of movement, nutrition as desired, and the full participation of support persons. While there are routine care processes in birth centers, such as intermittent auscultation of fetal heart tones and vital sign monitoring, there are no routine medical interventions, such as continuous external fetal monitoring, IV fluids, and continuous maternal vital sign monitoring.^21^

In the hospital‐based model of maternity care, care is largely provided by obstetricians (approximately 90% physician vs. 10% midwife) and births occur in hospitals.^22^, ^23^ Physician‐led hospital‐based management of pregnancy, labor, and birth varies across the United States, as evidenced by marked variation in the rates of intrapartum interventions and cesarean sections that is unexplained by clinical factors.^23^, ^24^, ^25^ Several aspects of the US healthcare system create pressure on hospital‐based providers that often leads to a culture of high intervention,^26^, ^27^ compounded by increasing rates of prepregnancy morbidity.^28^ While interventions can be life‐saving for women and infants with complex medical problems, admission to a hospital labor unit can also set off a cascade of interventions for low‐risk women; a recent multicenter evaluation of over 26,000 births found that over 90% of women with low‐risk pregnancies admitted in spontaneous labor received at least one intrapartum intervention.^24^

The objective of the current study is to evaluate the midwifery‐based birth center model of care as an integrated model of care for low‐risk pregnancies by comparing core birth outcomes of the midwifery‐based birth center model versus hospital‐based usual care. We included every element of the birth center model—those receiving midwifery‐based birth center care at labor onset who birthed in the birth center model, and those who intended birth center birth but transitioned to hospital‐based care during labor, birth, or postpartum. Thus, these data go beyond a comparison of birth outcomes by birth setting alone and allow a comparison of labor and birth outcomes by model of maternity care.

## 2 METHODS

### 2.1 Data

For the midwifery‐based birth center cohorts, we used the American Association of Birth Centers Perinatal Data Registry (AABC PDR) for maternal/infant observations collected between 2009 and 2019. This validated registry tracks outcomes for patients who initiated care at birth centers across the US and contains data detailing a patient's medical history, prenatal, intrapartum, and postpartum course.^29^, ^30^ Participation in the PDR is open to all birth centers in the US regardless of staffing or accreditation status. The decision to participate is determined at the practice level and is semi‐voluntary, as participation in a national midwifery data registry is a requirement for accreditation by the Commission for the Accreditation of Birth Centers (CABC). The AABC PDR has been determined to be exempt from review by the New England Institutional Review Board. Intrapartum and postpartum transfers from birth center care to a hospital, which occur in 12%–17% of birth center births, were included in the birth center cohort.^11^

**FIGURE A1:**
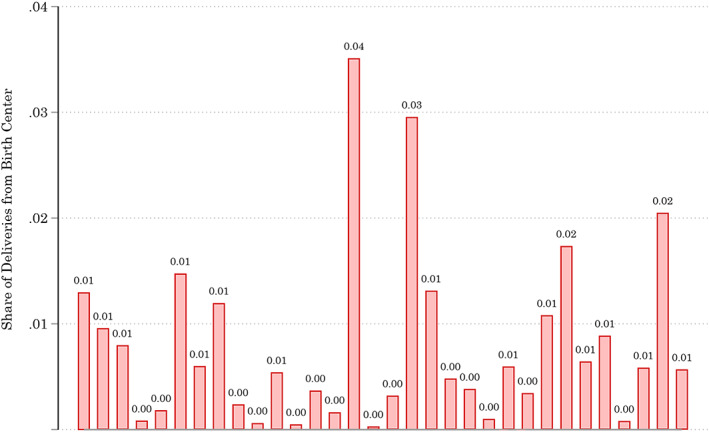
Amount of contamination of hospital‐based usual care cohort (birth certificate data), by state. [Color figure can be viewed at wileyonlinelibrary.com]

For a matched comparison group, we used the restricted access Natality Detail Files for 2009–2019 from the National Center for Health Statistics, Centers for Disease Control and Prevention. This data source reports all birth certificate records for all states in the US and includes characteristics of the mother, newborn, and delivery. The restricted data also report the mother's residence and the delivery location (state and county).^31^ The hospital‐based control group suffers from potential contamination, as birth center clients who ultimately gave birth in a hospital were included in both the registry data and birth certificate data. However, based on our evaluation of the data, we expect this contamination to be small, including only 1%–2% of all hospital deliveries (Figure A1).

### 2.2 Sample selection

The main analysis sample included women with low‐risk pregnancies, defined as a singleton fetus, no record of hypertension, no history of cesarean section, and maternal age between 16 and 45 years. Exclusion of other high‐risk pregnancy conditions was not possible due to limitations in birth certificate data. For both groups, we removed individuals who were missing key demographic information, with the most common missing elements being maternal education and maternal weight. The sample was also selected only for records that overlapped in each year and state. Counties were included only if there were at least 50 midwifery‐based birth center births from the AABC PDR and 300 total births. To account for regional variation in clinical management of pregnancy, we matched individuals within each county, using key maternal characteristics. See Table A1 for specific sample inclusion criteria.

Those receiving midwifery‐based birth center care at labor onset were included in the initial birth center sample (*N* = 88,141); those receiving hospital‐based usual care at labor onset were included in the initial hospital sample (*N* = 7,397,913). After nearest neighbor matching in eligible counties, the midwifery‐based birth center cohort sample was 85,842 and the hospital‐based model sample was 261,439. See Table 1 for baseline characteristics of the unmatched and matched cohorts. The final sample included 29 states and Washington, DC, with a state distribution of 10% in the Northeast, 31% in the South Atlantic, 14% in the South Central, 24% in the Midwest, and 24% in the West.

**TABLE 1:** Baseline characteristics of the unmatched and matched midwifery‐based birth center and hospital‐based usual care cohort.

	Unmatched		Matched	
Characteristic	Midwifery‐based birth center cohort *N* = 88,141 (%)	Hospital‐based usual care cohort *N* = 7,397,913 (%)	Midwifery‐based birth center cohort *N* = 85,842 (%)	Hospital‐based usual care cohort *N* = 261,439 (%)
Education, high school	15,954 (18.1)	1,753,305 (23.7)	15,537 (18.1)	46,275 (17.7)
Education, some college	17,540 (19.9)	1,997,437 (27.0)	16,997 (19.8)	53,856 (20.6)
Education, completed college	45,304 (51.4)	2,478,301 (33.5)	44,037 (51.3)	134,118 (51.3)
Payment, Medicaid	20,272 (23.0)	3,055,338 (41.3)	19,915 (23.2)	59,347 (22.7)
BMI, underweight	3702 (4.2)	466,069 (6.3)	3605 (4.2)	9935 (3.8)
BMI, overweight	7316 (8.3)	998,718 (13.5)	7125 (8.3)	20,654 (7.9)
BMI, obese	8990 (10.2)	1,479,583 (20.0)	8842 (10.3)	26,405 (10.1)
Diabetes	3173 (3.6)	392,089 (5.3)	3176 (3.7)	8366 (3.2)
Non‐Hispanic White	66,899 (75.9)	3,232,888 (43.7)	65,068 (75.8)	200,001 (76.5)
Non‐Hispanic Black	6170 (7.0)	924,739 (12.5)	6009 (7.0)	18,039 (6.9)
Hispanic	9607 (10.9)	2,485,699 (33.6)	9443 (11.0)	28,758 (11.0)
Maternal age	29.3	28.3	29.3	29.1
Parity	1.1	1.3	1.1	1.1

*Note*: Matching accomplished using propensity score weights (PSWs). PSWs were constructed by running a logistic regression, where the outcome is a binary variable capturing whether the individual participated in the midwifery‐based birth center model; the logistic regression controls for demographic characteristics and year indicators. After running the logistic regression, we predict the propensity scores in Stata (using predict, pr) and use these PSW to match PDR participants to their five nearest neighbors in the comparison group.

### 2.3 Statistical analysis

Consistent with previous comparable analyses, we used propensity scores to create matched comparison groups.^32^, ^33^ We then applied propensity weighting to the statistical analysis.

In our analysis, we constructed a within‐county comparison group using nearest neighbor matching for all women. We also matched by subgroupings of nulliparous (no previous childbirth) and multiparous (a history of at least one previous childbirth). This nearest neighbor matching selects a comparable hospital‐based comparison group, which is based on the observable characteristics of individuals. To perform the nearest neighbor matching, we ran separate logistic regressions for each county and each parity (nulliparous/multiparous). In these logistic regressions, the outcome was a binary variable capturing participation in the midwifery‐based birth center model. The logistic regressions included controls for demographic characteristics (self‐reported indicators for non‐Hispanic White, non‐Hispanic Black, and Hispanic; indicators for high school, some college, and completed college education; Medicaid enrollment and maternal age; medical risk factors including diabetes, BMI categories [overweight, underweight, or obese]) and year of birth indicators. Following these logistic regressions, we predicted the propensity score weights (PSWs, using the Stata module predict, pr). We then used these propensity scores to perform a nearest neighbor matching (using Stata's psmatch2) and selected the five closest individuals (based on observable characteristics) for the hospital‐based comparison group.

**FIGURE A2:**
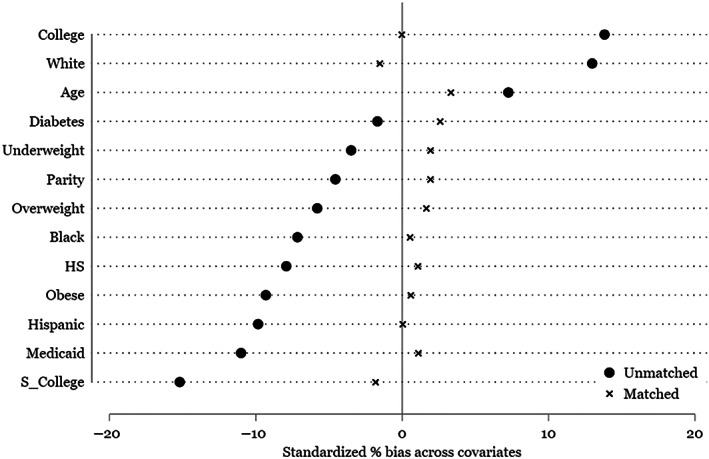
Balance across control variables after reweighting, hospital‐based usual care cohort.

These constructed PSWs (from the logistic regression) were then used as weights in the primary linear regression analysis. This linear regression analysis considers whether key maternity care outcomes differ across the maternity models of care. When applying PSW to the primary analysis, the estimates are reweighted to place a larger weight on individuals in the hospital‐based cohort who appeared most similar to the midwifery‐based birth center group. In the preferred specification, we also controlled for demographic characteristics and medical risk factors, including self‐reported indicators for non‐Hispanic White, non‐Hispanic Black, and Hispanic; indicators for high school, some college, and a completed college education; Medicaid enrollment and maternal age; medical risk factors including diabetes and BMI categories (overweight, underweight, or obese). Our controlled model also included year of birth indicators, indicators capturing the county of birth, and indicators for the match pair comparison. In the multipara sample, a control for parity (continuous) was added. The inclusion of other medical risk factors was not possible due to limited reporting in the restricted birth certificate records. Controls were also included for time‐varying state‐level policies, including CNM/CM independent practice regulation, CNM/CM collaborative practice regulation, and CPM licensing. After reweighting, the hospital‐based usual care cohort was balanced along control variables compared with the midwifery‐led birth center cohort to 3.5% (Figure A2). In all linear regressions, robust standard errors were clustered at the county level.

For our main analysis, we focused on the impact of the maternity care model (midwifery‐based birth center vs. hospital‐based usual care) as our key independent variable. The primary dependent variables, selected based on data availability, were the mode of delivery (cesarean section vs. vaginal), low birth weight, neonatal intensive care unit (NICU) admission, breastfeeding rates at discharge, and neonatal death.

### 2.4 Sensitivity analyses

We then performed several sensitivity analyses. First, we included results with alternative weighting methodologies. For these alternative weighting strategies, we present the same linear regressions using inverse probability weights (IPWs) and entropy balancing weights (EBWs) rather than PSW. The inverse probability weights (IPWs) were constructed similarly to the PSW, except that the IPW is 1/PSW for the midwifery‐based birth center group and [1/(1−PSW)] for the hospital‐based usual care group. For the EBWs, we used the Stata module ebalance, which addresses the problem of simultaneously matching on propensity scores and values of the explanatory variables in the propensity score equation. These matching methods address only the comparability of midwifery‐based birth centers and hospital‐based usual care groups on observed variables. Brooks and Ohsfeldt emphasize that when subjects voluntarily choose the treatment versus the control group (i.e., select into treatment), controlling for observed differences in the two groups of subjects increases the importance of unobserved differences.^34^ If those unobserved differences are correlated with the error term in the outcome equation, then matching can increase omitted variable bias.

To assess the importance of omitted variable bias in our analysis, we used a methodology developed by Oster.^35^, ^36^ This method assesses the degree of selection on observed factors relative to observable characteristics. The resulting Oster bound assesses the degree of selection on observables that would be required to explain away the observed effect of the midwifery‐based birth center model of care.

## 3 RESULTS

Table 2 shows differences across the key clinical outcomes for all women, and subgroups of nulliparous and multiparous women, using PSWs. Both nulliparous and multiparous women who received care in the midwifery‐based birth center model experienced lower rates of cesarean section, low birth weight, neonatal admission to the NICU, and neonatal death, and increased rates of breastfeeding at discharge compared with the hospital‐based usual care (comparison) group.

**TABLE 2:** Estimated differences in key birth outcomes across midwifery‐based birth center care and hospital‐based usual care using propensity score weights.

Column				
A	B	C	D	E
Outcome variables	Midwifery‐based birth center care	Hospital‐based usual care	Propensity score weights with controls	Propensity score weights with controls
	Mean (%)	Mean (%)	Difference (percentage points)	Confidence intervals
**(a) All women**				
Cesarean section	7.6	19.9	−12.2***	[−13.4, −11.1]
Low birth weight	1.9	5.2	−3.2***	[−3.6, −2.8]
NICU admission	1.1	6.7	−5.6***	[−6.5, −4.8]
Breastfeeding at discharge	97.4	87.5	9.3***	[6.9, 11.7]
Neonatal death	0.1	0.2	−0.1***	[−0.2, −0.1]
*N*	85,842	261,439		

(b) Nulliparous				
Cesarean section	14.6	27.8	−12.8***	[−14.2, −11.4]
Low birth weight	2.4	5.8	−3.2***	[−3.9, −2.6]
NICU admission	1.4	8.0	−6.6***	[−7.5, −5.7]
Breastfeeding at discharge	97.1	89.8	6.7***	[4.5, 9.0]
Neonatal death	0.1	0.2	−0.2***	[−0.3, −0.1]
*N*	36,258	102,853		

(c) Multiparous				
Cesarean section	2.6	14.7	−12.1***	[−13.3, −10.8]
Low birth weight	1.6	4.8	−3.2***	[−3.6, −2.7]
NICU admission	1.0	5.9	−4.9***	[−6.0, −3.8]
Breastfeeding at discharge	97.6	86.0	11.2***	[8.3, 14.1]
Neonatal death	0.1	0.2	−0.2***	[−0.2, −0.1]
*N*	49,584	158,586		

*Note*: [1] Columns (B) and (C) include the unweighted means across groups. [2] Estimates reflect a linear regression analysis where propensity score reweighting (PSW) has been applied. PSWs are constructed by running a logistic regression, where the outcome is a binary variable capturing whether the individual participated in a midwifery‐based birth center model; the logistic regression controls for demographic characteristics and year indicators. After running the logistic regression, we predict the propensity scores in Stata (using predict, pr) and use these PSW to match PDR participants to their five nearest neighbors in the comparison group. [3] Models with covariates include controls for age, education category, BMI category, Medicaid status, diabetes status, race, and controls for time‐varying state‐level policies, including CNM/CM independent practice, CNM/CM collaborative practice, and CPM licensing. Columns with controls also include indicators for the year of birth, indicators for the county, and indicators for matched pair. Models with multiparas also include parity as a control. [5] Robust standard errors are clustered at the county level.

*** , **, and * represent statistical significance at 1%, 5%, and 10% levels, respectively.

The average cesarean section rate for women receiving care in the birth center model was 7.6% versus the comparison group cesarean section rate of 19.9% (*p* < 0.001). Low birth weight in the birth center cohort occurred in 1.9% versus 5.2% in the comparison group (*p* < 0.001). NICU admission followed a similar pattern. Infants of women receiving care in the birth center model were admitted to the NICU at a rate of 1.1% versus 6.7% in the comparison group (*p* < 0.001).

Rates of breastfeeding at discharge were higher in the cohort receiving care in the birth center model, with an average breastfeeding rate of 97.4% versus 87.5% for the comparison group (*p* < 0.001). Neonatal death was infrequent, regardless of intended birth setting; neonatal death was 0.1% in the birth center cohort versus 0.2% in the comparison group (*p* < 0.001).

Notable differences in the nulliparous versus multiparous subgroups include differences in the cesarean section rate. The average cesarean section rate for nulliparas receiving care in the midwifery‐based birth center model was 14.6% compared to 27.8% in the comparison group, a point estimate that suggests a cesarean section rate for nulliparas that is 48% lower in the midwifery‐based birth center group than the comparison group. For multiparas, the cesarean section rate was 2.6% for the birth center model cohort versus a cesarean section rate of 14.7% for the comparison group (*p* < 0.001), where the point estimate suggests an 82% lower risk for cesarean section among multiparas in the birth center model.

We then performed two sensitivity analyses using IPWs and EBWs (see Table 3). The results in outcome measures were consistent across methods.

**TABLE 3:** Estimated differences in key birth outcomes across midwifery‐based birth center care and hospital‐based usual care for alternate weighting methods.

Column								
A	B	C	D	E	F	H	I	J
Outcome variable	Midwifery‐based birth center care	Hospital‐based usual care	Propensity score weights	Propensity score weights with controls	Inverse probability weights	Inverse probability weights with controls	Entropy balancing weights	Entropy balancing weights with controls
	Mean %	Mean %	Difference (percentage points) [CI]	Difference (percentage points) [CI]	Difference (percentage points) [CI]	Difference (percentage points) [CI]	Difference (percentage points) [CI]	Difference (percentage points) [CI]
**All women**								
Cesarean section	7.6	19.9	−12.3*** [−13.4, −11.2]	−12.2*** [−13.4, −11.1]	−13.1*** [−14.7, −11.6]	−12.6*** [−13.8, −11.5]	−12.1*** [−13.6, −10.7]	−12.5*** [−13.8, −11.3]
Low birth weight	1.9	5.2	−3.0*** [−3.4, −2.6]	−3.2*** [−3.6, −2.8]	−3.5*** [−3.9, −3.1]	−3.3*** [−3.8, −2.9]	−3.1*** [−3.6, −2.6]	−3.2*** [−3.7, −2.7]
NICU admission	1.1	6.7	−5.6*** [−6.3, −4.8]	−5.6*** [−6.5, −4.8]	−5.9*** [−6.6, −5.2]	−5.7*** [−6.5, −4.9]	−5.6*** [−6.8, −4.4]	−5.6*** [−6.8, −4.5]
Breastfeeding at discharge	97.4	87.5	9.0*** [6.6, 11.4]	9.3*** [6.9, 11.7]	10.7*** [8.2, 13.1]	9.8*** [7.4, 12.2]	9.8*** [6.8, 12.8]	9.5*** [6.9, 12.2]
Neonatal death	0.1	0.2	−0.1*** [−0.2, −0.1]	−0.1*** [−0.2, −0.1]	−0.1*** [−0.2, −0.0]	−0.1*** [−0.2, −0.1]	−0.2*** [−0.3, −0.1]	−0.2*** [−0.2, −0.1]
*N*	85,842	261,439						

*Note*: [1] Columns (B) and (C) include the unweighted means across groups. [2] Estimates reflect a linear regression analysis where different weighting schemes (PSW/IPW/EBW) have been applied. PSWs are constructed by running a logistic regression, where the outcome is a binary variable capturing whether the individual participated in a midwifery‐led birth center—the logistic regression controls for demographic characteristics and year indicators. After running the logistic regression, we predict the propensity scores in Stata (using predict, pr) and use these PSWs to match PDR participants to their five nearest neighbors in the comparison group. The inverse probability weights (IPWs) were constructed similarly to the PSW, except the IPW is 1/PSW for the midwifery‐led birth center group and [1/(1−PSW)] for the hospital‐based usual care group. For the entropy balancing weights, we used the Stata module ebalance, which addresses the problem of simultaneously matching on propensity scores and values of the explanatory variables in the propensity score equation. [3] Models with covariates include controls for age, education category, BMI category, Medicaid status, diabetes status, race, and controls for time‐varying state‐level policies, including CNM/CM independent practice, CNM/CM collaborative practice, and CPM licensing. Columns with controls also include indicators for the year of birth, indicators for the county, and indicators for matched pair. Models with multiparas also include parity as a control. [5] Robust standard errors clustered at the county level.

*** , **, and * represent statistical significance at 1%, 5%, and 10% levels, respectively.

Last, we explored omitted variable bias using Oster's methodology (Table 4).^35^, ^36^ In this bounding methodology, all computed confidence intervals show a similar range of estimates to the main effect. Consistent with Oster (2019) who notes that if the bounds exclude zero and delta is above one, then the results are robust to omitted variable bias; this sensitivity analysis suggests that the influence of omitted variable bias is small.

**TABLE 4:** Propensity score weight estimated difference across midwifery‐based birth center care and hospital‐based care with Oster bounding to assess omitted variable bias.

	Cesarean—PSW	Low birth weight—PSW	NICU—PSW	Breastfeeding—PSW	Death—PSW
	b/se	b/se	b/se	b/se	b/se
**(a) All women**					
Midwifery‐based birth center	−12.2***	−3.2***	−5.6***	−0.1***	−0.1***
	0.6	0.2	0.4	1.2	0.0
Observations	347,236	335,591	347,073	305,926	346,988
Degree of selection (delta)	2.926	5.982	2.976	2.707	6.179
Upper bound	−12.3	−3.0	−5.6	9.0	−0.1
Lower bound	−12.2	−3.4	−5.7	9.8	−0.2
Adjusted *R* ^2^	0.299	0.263	0.271	0.310	0.248

(b) Nulliparous					
Midwifery‐based birth center	−12.8***	−3.2***	−6.6***	6.7***	−0.2***
	0.7	0.3	0.5	1.2	0.1
Observations	139,090	134,409	139,030	120,566	138,981
Degree of selection (delta)	2.678	3.324	2.759	2.485	−2.440
Upper bound	−12.6	−3.1	−6.5	6.3	−0.1
Lower bound	−12.9	−3.4	−6.8	7.4	−0.2
Adjusted *R* ^2^	0.387	0.373	0.379	0.386	0.303

(c) Multiparous					
Midwifery‐based birth center	−12.1***	−3.2***	−4.9***	11.2***	−0.2***
	0.6	0.2	0.5	1.5	0.0
Observations	208,146	201,182	208,043	185,360	208,007
Degree of selection (delta)	3.565	5.954	3.484	3.364	2.448
Upper bound	−12.0	−3.0	−4.9	10.9	−0.2
Lower bound	−12.2	−3.3	−4.9	11.6	−0.1
Adjusted *R* ^2^	0.396	0.371	0.373	0.422	0.385

*Note*: The table shows PSW estimates from the main table and Oster bounds based on Oster's (2019) methodology. While we match individuals with a suitable control group based on observables, unobservable factors may lead individuals to choose AABC PDR birth centers, and these unobservables would conflate our estimated treatment effect. To assess this concern, we follow Oster (2019) and show reasonable bounds on our estimates. Following our linear regression, we calculate these bounds using Oster's Stata package pscalc. We assume an Rmax of 1.3 times the R‐squared in the controlled linear regression. Oster (2019) notes that if the bounds exclude zero, and delta is above one, the results are robust to omitted variable bias.

*** , ** and * represent statistical significance at 1%, 5% and 10% levels, respectively.

## 4 DISCUSSION

In this national sample comparing matched women with low‐risk pregnancies and births, both nulliparous and multiparous women who received care in the midwifery‐based birth center model (vs. hospital‐based usual care) experienced lower rates of cesarean section, low birth weight, NICU admission and neonatal death, and increased rates of breastfeeding at discharge, regardless of where birth ultimately occurred. Of note, NICU admission among the cohort birthing in a hospital may be a signal of proximity to the NICU or an in‐hospital culture favoring NICU observation of essentially healthy neonates rather than a true signal of risk difference.^37^, ^38^

Notably, in the midwifery‐based birth center cohort, the cesarean section rate for nulliparous women was reduced by 48% and the rate for multiparous women was reduced by 82% when compared with hospital‐based usual care. These results were supported by two sensitivity analyses that accounted for differences in observable characteristics and one sensitivity analysis accounting for unobservable characteristics.

Our study results are consistent with existing evidence that midwifery‐based birth center care is associated with lower rates of medical interventions and higher rates of breast feeding.^7^ A similar evaluation of the integrated midwifery‐based birth center model, generated from the federal Strong Start for Mothers and Newborns Initiative, found significantly lower rates of low birth weight, preterm birth, and cesarean birth in a national cohort of Medicaid recipients.^33^ However, our analysis differs from other published research using birth certificate data that suggests an increased risk of poor neonatal outcomes in birth center births.^39^, ^40^

Limitations of the current study include the general limitations of birth certificate and registry data. Although birth certificate data quality for demographic variables is good, accuracy for medical variables is of less quality.^7^ In addition to data quality concerns, most birth certificate data lack information on intentionality, or where an individual intended to give birth at the start of labor. Therefore, individuals in the birth center cohort who were transferred intrapartum and gave birth in a hospital were included in birth certificate data as well as in registry data. Our analysis indicates this contamination is only 1%–2% of the hospital‐based sample. In addition, we were limited in our ability to determine the care provider after transfer; care may have continued in the birth center model, provided by birth center midwives, or care may have transitioned to the standard hospital‐based model, provided by hospital‐affiliated providers. Any birth center provider‐related contamination is likely to be small, as fewer than one third of birth centers have hospital privileges.^10^ Any contamination of this kind, however, would bias the results toward no significant difference between the two groups.

In addition, birth certificate records limit the reporting of midwifery birth attendant to either “CNM/CM” or “other midwife.” Moreover, birth certificates are known to substantially underreport CNM/CM attended births, and in cases of cesarean section, the attendant will always be listed as physician.^41^, ^42^ These limitations make it difficult to control for, or separate by, midwife certification (CNM, CM or CPM) in this study, because the information will only be available in the AABC PDR and not in our comparison group from the birth certificate records.

There are limitations to registry data as well. The AABC PDR includes outcomes of care provided by all categories of midwives. It is currently unknown whether there are differences in outcomes based on type of midwife certification, although the training and educational requirements of all three categories of midwives in the US conform to the International Confederation of Midwives Global Standards for Midwifery Education.^14^ However, participation in the PDR is decided at the practice level, so all outcomes for all clients of the practice who provide consent are included, preventing selective exclusion of cases from certain providers or those with poor outcomes. AABC and CABC hold all birth centers to the same standards, regardless of midwife credentialing.

Another limitation to the AABC PDR is that birth centers that contribute, although geographically diverse, are self‐selected, and may not be representative of all US birth centers. Any maternity care provider in the US is eligible to participate in the PDR regardless of provider type or practice setting; however, for birth centers accredited by CABC, participation in a national midwifery data registry is required.^43^ Thus, while only about half of US birth centers are accredited,^10^ the majority of those contributing to the AABC PDR are accredited. These factors may skew the PDR toward a subset of “high‐quality” birth centers, which may limit the generalizability of findings to all US birth centers.

The strengths of our analysis include our robust methods of statistical analysis, as analyses of observational data using propensity score methods better approximate randomized controlled trials than traditional statistical approaches.^44^, ^45^ An additional strength is the use of a validated data source for birth center outcomes. The PDR has been shown to be highly reliable, with over 97% consistency across variables.^30^ One difficulty of research on midwifery care in the United States is that regulation of birth centers and midwives varies greatly by state, and regulations can help or hinder integration of midwifery.^12^ We were able to reduce the effect of these variable regulations by controlling for state‐level policies, including CNM/CM independent practice regulation, CNM/CM collaborative practice regulation, and CPM licensing.^46^ Another limitation of the study of midwifery‐based birth center care is the concern that women selecting into birth centers may be different in some way from those receiving hospital‐based usual care, and those differences affect both outcomes and the delivery locations. We accounted for observable characteristics through our use of propensity score matching and weighting. To account for unobservable characteristics, we relied on the Oster ratios, which indicate how large the selection on unobservable characteristics would have to be to overcome the selection on observables. The Oster ratios in our analysis suggest that the influence of unobservable characteristics on our results may be small.

A critical strength was our ability to assess midwifery‐based birth center care as an integrated model of care, as we were able to include birth outcomes for clients who transferred from birth center to hospital. Recently, particular attention has been paid to the impact of birth setting and provider type on birth outcomes.^7^ However, considering these factors in isolation is problematic.^47^ Birth outcomes are impacted by complex systems of care that are shaped by multiple interrelated factors. While this includes provider type and setting, it also includes factors that may be more challenging to measure, such as the composition of maternity care teams, culture of care, or level of interprofessional collaboration.^48^, ^49^, ^50^, ^51^, ^52^ The current study builds upon existing research and offers a broader examination of systems of maternity care, advancing our understanding of an optimal maternity care system.

Worsening maternal outcomes and increasing maternal health disparities in the United States clearly indicate the need for systems change.^53^, ^54^ Successfully managing the divergent needs of high‐risk and low‐risk pregnancies will require a remodeling of our maternity care system toward one that matches care model to individual patient needs and has the flexibility to pivot when patient needs change.^55^ Expanding the availability of integrated midwifery‐based birth center birth offers a path to improve care for low‐risk maternal/newborn dyads. Worldwide, numerous high‐quality, country‐level integrated maternity care systems exist as exemplars, with multiple strategies for matching care provider type to individual patient risk level across a range of birth settings.^56^, ^57^ Our study supports lower rates of cesarean section, low birth weight, NICU admission, and neonatal death, along with increased rates of breastfeeding, for women utilizing midwifery‐based birth center care. These findings are consistent with existing international evidence that integrated midwifery‐based birth center care delivers improved outcomes for women at low risk for perinatal complications.^58^, ^59^, ^60^

## FUNDING INFORMATION

No funding to report.

## APPENDIX A

**TABLE A1:** Covariates and sample selection for the main analysis.

Covariates	Exclusions	Locational Selection
Education (indicators for high school degree, some college, and college educated)	Missing any key information; including covariates, parity, place of birth, attendant	Birth certificates: Hospital deliveries only
BMI (indicators for obese, overweight, and underweight)	Outside the ages of 16–45	Midwifery‐led birth center: All delivery locations
	Any hypertension	States with at least 50 birth center deliveries and year‐state combinations with at least one birth center delivery
Race/ethnicity (indicators for White, Black, and Hispanic)	Non‐singleton	
	Previous Cesarean	
Age (continuous)		Counties with at least 50 birth center deliveries and 300 hospital birth certificate deliveries
Medicaid payer (indicator)		
Parity (continuous)		
